# PET bottle recycling system in Asia: Lessons learned from six cities

**DOI:** 10.1016/j.isci.2026.118095

**Published:** 2026-11-02

**Authors:** Xuemei Zhang, Faith Ka Shun Chan, Yuanyuan Tang, Yuyao Xu, Chao Wang, Matthew Johnson, Taiseer Nafea, Yaoyang Xu, Yong-Guan Zhu, Wei-Qiang Chen

**Affiliations:** 1 School of Geographical Sciences, University of Nottingham Ningbo China, Ningbo 315100, China; 2 Water@Leeds and School of Geography, University of Leeds, LS2 9JT Leeds, UK; 3 State Key Laboratory of Regional and Urban Ecology, Institute of Urban Environment, Chinese Academy of Sciences, Xiamen 361021, China; 4 College of Economics and Management, Beijing University of Technology, Beijing, China; 5 School of Geography, University of Nottingham, University Park, NG7 2RD Nottingham, UK

**Keywords:** post-consumer PET bottles, Asian cities, comparative analysis, waste management, recycling

## Abstract

The rapid growth of bottled water consumption has increased post-consumer polyethylene terephthalate (PET) bottle waste, while the recycling performance varies substantially across Asian cities. This study comparatively examines PET bottle recycling systems in Tokyo, Seoul, Shanghai, Hong Kong, Bangkok, and Kuala Lumpur, focusing on policy and governance, producer and recycler practices, recycling technologies, market conditions, and informal recycling actors. The results reveal distinct recycling pathways: Tokyo and Seoul have mature policy systems and are advancing high-value recycling. Shanghai has a dynamic market-led system dominated by downcycling. Hong Kong shows gradual policy improvement but limited effectiveness, and Bangkok and Kuala Lumpur remain highly reliant on informal recycling under weak enforcement. These differences highlight the importance of coordinated policy, market, technology, and social actors in shaping urban PET recycling outcomes. The study identifies transferable practices, including source separation, inclusive integration of informal recyclers, extended producer responsibility, and effective government coordination, providing guidance for improving PET recycling systems across Asia.

## Highlights

• Six Asian cities have diverse PET bottle recycling systems
• Tokyo and Seoul have mature recycling systems and are exploring chemical recycling
• Shanghai has a vibrant market-driven recycling system but mostly downcycles PET bottles
• With limited governance, informal recyclers, communities, and pilots play key roles

## Graphical abstract

**Figure:**
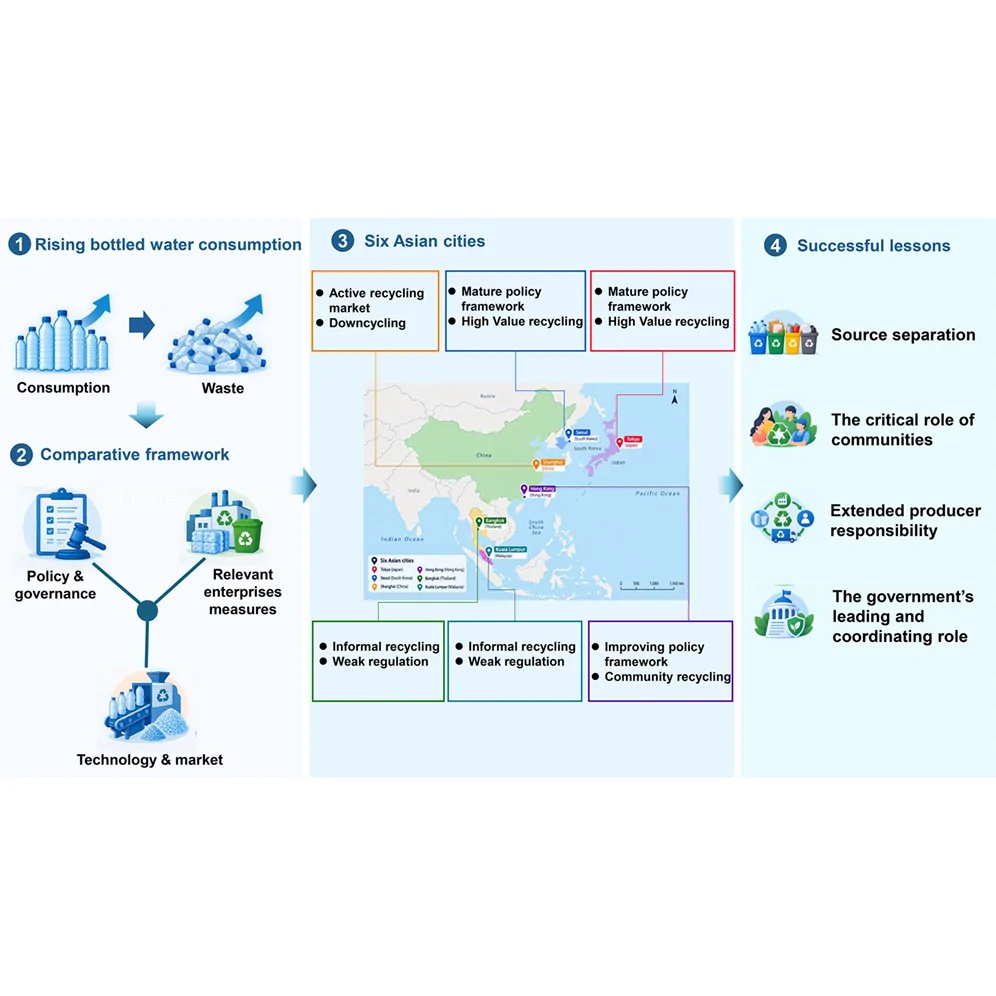

## Teaser

Environmental science; Environmental issues

## Introduction

Growing concerns over drinking water safety have accelerated global bottled water consumption and, consequently, the demand for polyethylene terephthalate (PET) bottles. In 2025, the global PET bottle market reached 26.23 million tons, and it is projected to increase to 33.49 million tons by 2031, with a compound annual growth rate of 4.15% during 2026–2031.^1^ This growth is particularly pronounced in emerging economies, with the Asia Pacific region contributing the largest absolute increase in bottled water consumption.^2^ Yet PET bottle recycling remains far from sufficient. In Southeast Asia, the average recycling rate is only about 26%.^3^ Where recycling infrastructure is weak, discarded PET bottles are often landfilled, incinerated, or leaked into the environment, causing resource losses and increasing pressure on marine ecosystems.^4^ Improving PET bottle circularity is becoming an urgent issue in plastic governance. However, global governance remains constrained by disagreements over responsibility allocation and implementation pathways, as reflected in the stalled negotiations of the United Nations plastic treaty.^5^^,^^6^

Regulatory pressure and corporate sustainability commitments are reshaping the demand for recycled PET. Major beverage companies, including Danone, Coca-Cola, PepsiCo, and Suntory, have pledged to increase recycled PET content in packaging to 25%–100% between 2025 and 2030. These commitments make Asia particularly important to the future of PET bottle circularity, not only because it is a major consumption region but also because it serves as a key hub for recycling activities and secondary material flows. Since PET bottle recovery is ultimately implemented through urban collection, sorting, and recycling systems, city-level governance and infrastructure play a critical role in determining recycling performance. A higher level of green development can further enhance economic stability and sustainability, providing stronger institutional and material foundations for improving PET bottle recycling systems.^7^

Existing research has provided important insights into PET recycling technologies, including mechanical and chemical recycling technologies,^8^ environmental impact assessments,^9^^,^^10^^,^^11^ and post-recycling applications.^12^^,^^13^^,^^14^ However, much less attention has been paid to how PET bottle recycling systems are governed, organized, and implemented in different urban contexts. Broader studies on plastic waste management have also tended to treat plastics as a single category,^15^^,^^16^^,^^17^ overlooking the material-specific features of PET bottles, such as their relatively high recyclability and market value. In addition, studies on developing regions, especially Southeast Asia,^18^^,^^19^^,^^20^ are often region-specific or limited to bilateral comparisons.^5^^,^^21^ Existing literature also tends to focus either on policy design^22^^,^^23^ or informal recycling sectors,^24^^,^^25^ while the role of enterprises and cross-actor coordination remains underexplored. To address these gaps, this study examines PET bottle circularity from a city-level governance perspective. Cities are critical arenas for recycling transitions because many collection systems, pilot programs, corporate initiatives, and public participation mechanisms are implemented locally. This study compares six Asian cities: Tokyo, Seoul, Shanghai, Hong Kong, Kuala Lumpur, and Bangkok. These cities all face high PET bottle consumption but differ substantially in economic development, governance capacity, recycling infrastructure, policy maturity, and market organization. They therefore provide a useful basis for comparing different PET bottle recycling pathways across advanced, transitional, and developing urban contexts. Their contextual information can be found in Table 1. Through a comparative analysis of these cities, this study aims to identify the key characteristics, constraints, and developmental patterns of PET bottle circularity across different urban contexts and to generate policy-relevant insights for other Asian cities facing similar conditions. Specifically, the objectives of this study are as follows:

(1) Systematically examine the PET bottle recycling systems in six Asian cities, with particular attention to municipal policies and their implementation measures.
(2) Examine how upstream packaging companies and downstream recyclers contribute to PET bottle recycling across different cities by comparing their recycling practices, technologies, and market condition.
(3) Analyze the major challenges and emerging opportunities encountered by each city in developing PET bottle recycling and broader circular systems.
(4) Identify best practices and transferable experiences that may provide insights for other Asian cities.

**Table 1:** Contextual information of the six focus cities in 2024

City	Population (million)	GDP per capita (USD)	Household waste generation (million metric tons)
Tokyo	13.91^26^	54,459^27^	3.79^28^
Seoul	9.59^29^	36,857^30^	3.81^30^
Shanghai	24.80^31^	31,490^31^	12.60^32^
Hong Kong	7.51^33^	54,300^33^	3.97^34^
Bangkok	10.91^35^	20,721^36^	3.10^37^
Kuala Lumpur	2.06^38^	30,679^38^	0.84^39^

## Results

### PET bottle recycling status of six cities in Asia

#### PET bottle recycling system and municipal intervention

The comparison of PET bottle recycling systems across the six cities was guided by the analytical framework presented in Table 2. The six cities reveal a clear institutional gradient in PET bottle recovery systems, ranging from highly formalized government-led systems to more fragmented market-mediated recovery networks. Tokyo and Seoul represent the most institutionalized models, where PET bottle recycling is embedded in national regulatory frameworks and translated into routine municipal collection practices. In Tokyo, the Waste Management and Public Cleansing Law and the Containers and Packaging Recycling Law^40^ provide the legal basis for mandatory separation and extended producer responsibility (EPR),^41^ while residents are required to rinse PET bottles and remove caps and labels before disposal. Seoul follows a similarly rule-based pathway,^42^ with transparent PET bottles collected separately from other plastics in apartment complexes and through dedicated weekly collection in detached housing areas.^43^ In both cities, front-end source separation functions as a quality-control mechanism, reducing contamination and enabling higher-value downstream recycling. Hong Kong also follows a government-supported pathway, but its system is less coercive and more strongly dependent on community-based and incentive-driven participation.^44^^,^^45^ The GREEN@COMMUNITY network, mobile collection points, smart recycling bins, and reverse vending machines (RVMs) have created a visible collection infrastructure,^46^ yet the recovery of PET bottles remains constrained by voluntary participation.^47^ These three cities exhibit predominantly government-led PET bottle recycling systems (as shown in Figure 1). From an institutional economics perspective, such systems reflect the capacity of public institutions to define responsibilities, reduce coordination uncertainty, and lower the transaction costs associated with collection, sorting, and downstream recycling.^48^^,^^49^ Public participation in PET bottle recycling depends less on spontaneous market incentives and more on formal rules, administrative coordination, and municipally supported infrastructure. This institutionalized pathway is particularly evident in Tokyo and Seoul, where refined source separation at the front end improves the purity of discarded PET bottles and enhances downstream recycling efficiency (Table 3). These advantages help explain their relatively strong PET bottle recycling performance. Detailed information on collection facilities, disposal instructions, and operational arrangements is provided in Table S1.

**Table 2:** Analytical framework for comparing PET bottle recycling systems

Analytical module	Analytical dimension	Evaluation focus	Purpose
Recycling system and municipal intervention	recycling model	• government-led recycling • market-led recycling • mixed recovery pathway	identify the dominant institutional pathway
	collection method	• source separation • collection points • mobile collection • informal collection	assess front-end collection quality
	policy instruments	• EPR • waste-separation rules • recycled-content targets • subsidies	evaluate operational capacity
	recycling infrastructure	• collection sites • transfer stations • sorting facilities • recycling centers • platform-based services	assess the role and effectiveness of municipal governance
Corporate practices and recycling market	packaging design and recycled PET use	• lightweight design • recyclable packaging • label design • recycled PET content	evaluate corporate contribution to high-quality recycling
	corporate recycling initiatives	• take-back schemes • reverse vending machines • bottle-to-bottle projects • recycling investment	assess enterprise participation beyond production
	recycling technology capacity	• mechanical recycling • chemical recycling • bottle-to-bottle recycling • sorting technologies	examine downstream processing capacity
	recycling market conditions	• recycling enterprise networks • recycled PET bottle quality • recycled PET demand market	assess market support for PET bottle recovery

**Figure 1:**
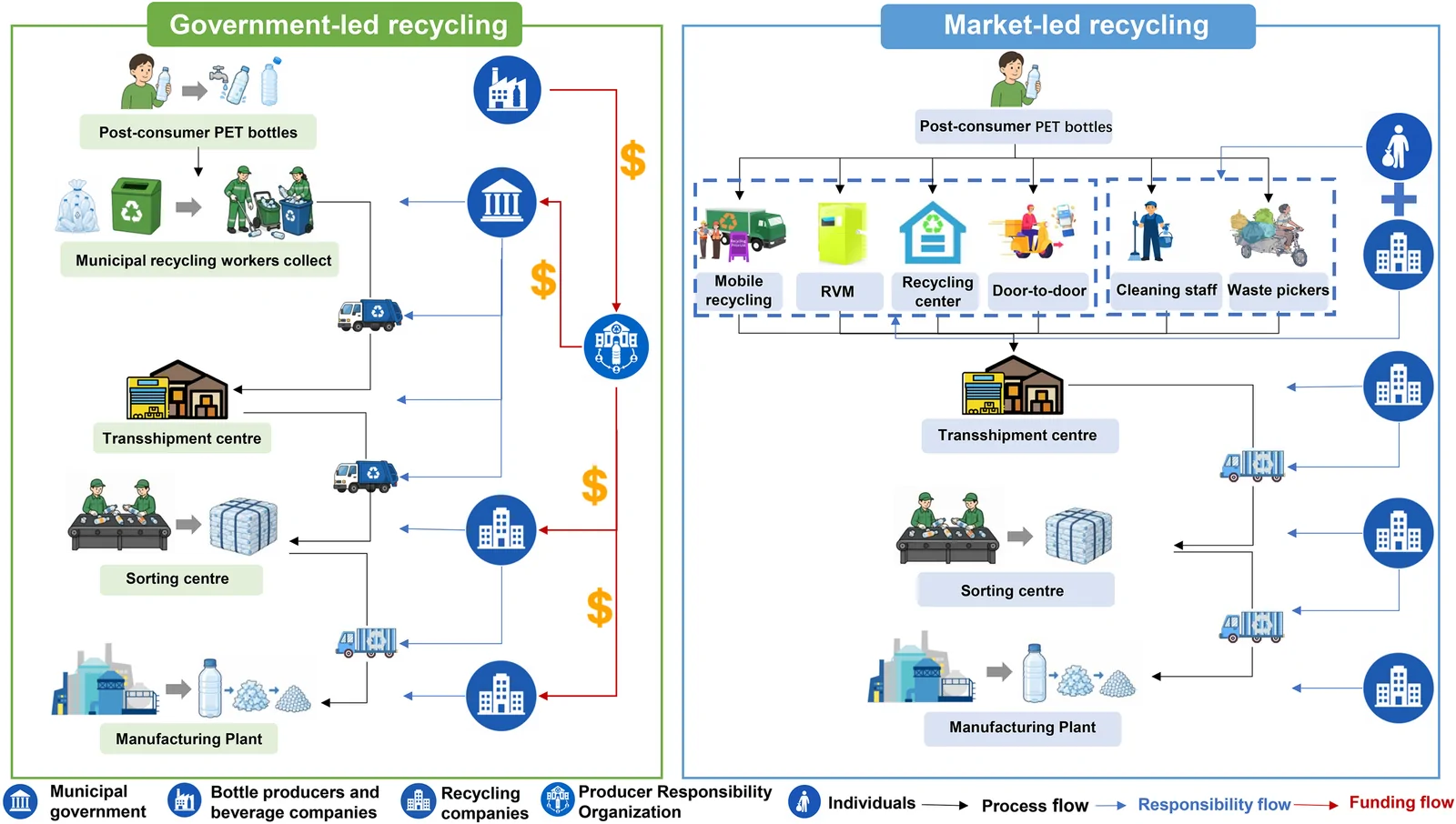
Comparison of government-led and market-led PET recycling systems The left panel illustrates a government-led PET bottle recycling model, in which municipal governments play a central role in organizing collection and recycling activities. The right panel illustrates a market-led model, in which recycling activities are primarily driven by market actors, including recycling enterprises and informal waste pickers.

**Table 2:** Comparison of PET bottle recycling system in six cities

City	Governance model	EPR	Recycling process participants	Municipal intervention effectiveness	Corporate practice efficiency	PET bottle recycling rate
Tokyo	government led	yes	• municipal government • bottle producers and beverage companies • recycling companies • producer responsibility organization • consumer	high	high	92%^50^
Seoul	government led	yes	• municipal government • bottle producers and beverage companies • recycling companies • producer responsibility organization • consumer	high	high	79%^42^
Hong Kong	government led	under development	• municipal government • recycling companies • informal collector	moderate	moderate	14%^51^
Shanghai	market led	no	• recycling companies • informal collector	moderate	high	95.4%^52^
Bangkok	market led	no	• recycling companies • informal collector	low	moderate	52%^53^
Kuala Lumpur	market led	no	• recycling companies • informal collector	low	low	23%^53^

Given the limited availability of city-level recycling rate data, multiple data sources were systematically compiled and subjected to uncertainty analysis and cross-validation. The most reliable estimates were then selected to represent the recycling rates of each city in 2024.

By contrast, Shanghai, Bangkok, and Kuala Lumpur are more strongly shaped by market-led recovery. Shanghai is moving from an informal-collector-based system toward an enterprise-led recovery network, supported by door-to-door collection, fixed-source collection, smart collection machines, and mobile recovery stations.^54^ Bangkok remains heavily dependent on informal collectors operating across households, collection streams, and disposal sites,^31^^,^^55^ while Kuala Lumpur relies on a combination of limited formal collection, household self-delivery, collection points, and scrap dealers. This pattern can be interpreted as a market-based response to gaps in formal waste governance. Where municipal capacity, fiscal resources, or regulatory enforcement are insufficient to support comprehensive public collection systems, the residual economic value of PET bottles creates incentives for private collectors, traders, recyclers, and informal actors to undertake recovery activities. Previous studies on waste management in developing countries similarly show that informal or market-oriented recycling networks often emerge where formal municipal systems are incomplete, providing a flexible but uneven substitute for public service provision.^56^ However, compared with government-led systems, market-driven recycling tends to prioritize materials with higher resale value, while system stability, data transparency, labor protection, and front-end quality control may remain weaker. From the perspective of polycentric environmental governance, these divergent pathways suggest that PET bottle circularity is not determined by a single governance mechanism but is instead shaped by the interactions among municipal governments, market actors, communities, and recycling enterprises under specific local institutional conditions.^57^

Policy intervention differs not only in intensity but also in the way municipal authority structures the behavior of residents, producers, and recycling enterprises. Tokyo and Seoul use the most comprehensive policy portfolios. Tokyo combines EPR decentralized municipal collection responsibilities,^58^ design guidelines for designated PET bottles,^59^ in-store take-back schemes,^60^ municipal subsidies, and recycled-content requirements,^61^ including the national requirement for PET beverage bottles to contain at least 15% recycled content from 2026. Seoul has similarly strengthened PET bottle recovery through producer fees, packaging recyclability grading, market restrictions on colored PET bottles, mandatory separation of transparent PET bottles,^62^ and financial support for unmanned PET collection machines.^42^ These instruments do not simply increase collection volume; they actively shape material quality, producer behavior, and downstream demand. Hong Kong has introduced a broad set of policy initiatives, including the Waste Blueprint for Hong Kong 2035,^63^ GREEN$ rewards,^64^ household plastic waste collection trials,^65^ and RVM pilots.^46^ The pilot has shown technical feasibility and a degree of public acceptance, but its system-wide contribution remains limited when benchmarked against Hong Kong’s overall plastic bottle consumption. In 2024, Hong Kong used an estimated 1.6 billion plastic bottles, whereas around 64.5 million bottles were collected through 120 RVMs, equivalent to only about 4% of total bottle use.^66^ Hence, although policy intervention has increased, its intensity remains insufficient relative to the scale of plastic waste generated. Shanghai occupies an intermediate position. Its policy instruments have not replaced the market-based recovery system, but they have increasingly formalized and upgraded it through plastic pollution control measures,^67^ recyclables system guidelines,^68^ transfer-station planning,^69^ mandatory waste sorting, and digital governance tools.^70^^,^^71^ In Bangkok and Kuala Lumpur, policy instruments remain comparatively weak or unevenly enforced. Bangkok has promoted 3R programs,^72^ zero-waste pilot districts,^73^ RVM pilot,^74^ and “Send Plastic Home” project,^75^ while Kuala Lumpur has introduced source-separation requirements under Malaysia’s Separation at Source Initiative.^76^ Nevertheless, limited enforcement capacity, insufficient incentives, and inadequate collection infrastructure continue to restrict the effectiveness of these measures.^77^ Figure 2 shows the policies adopted by each city.

**Figure 2:**
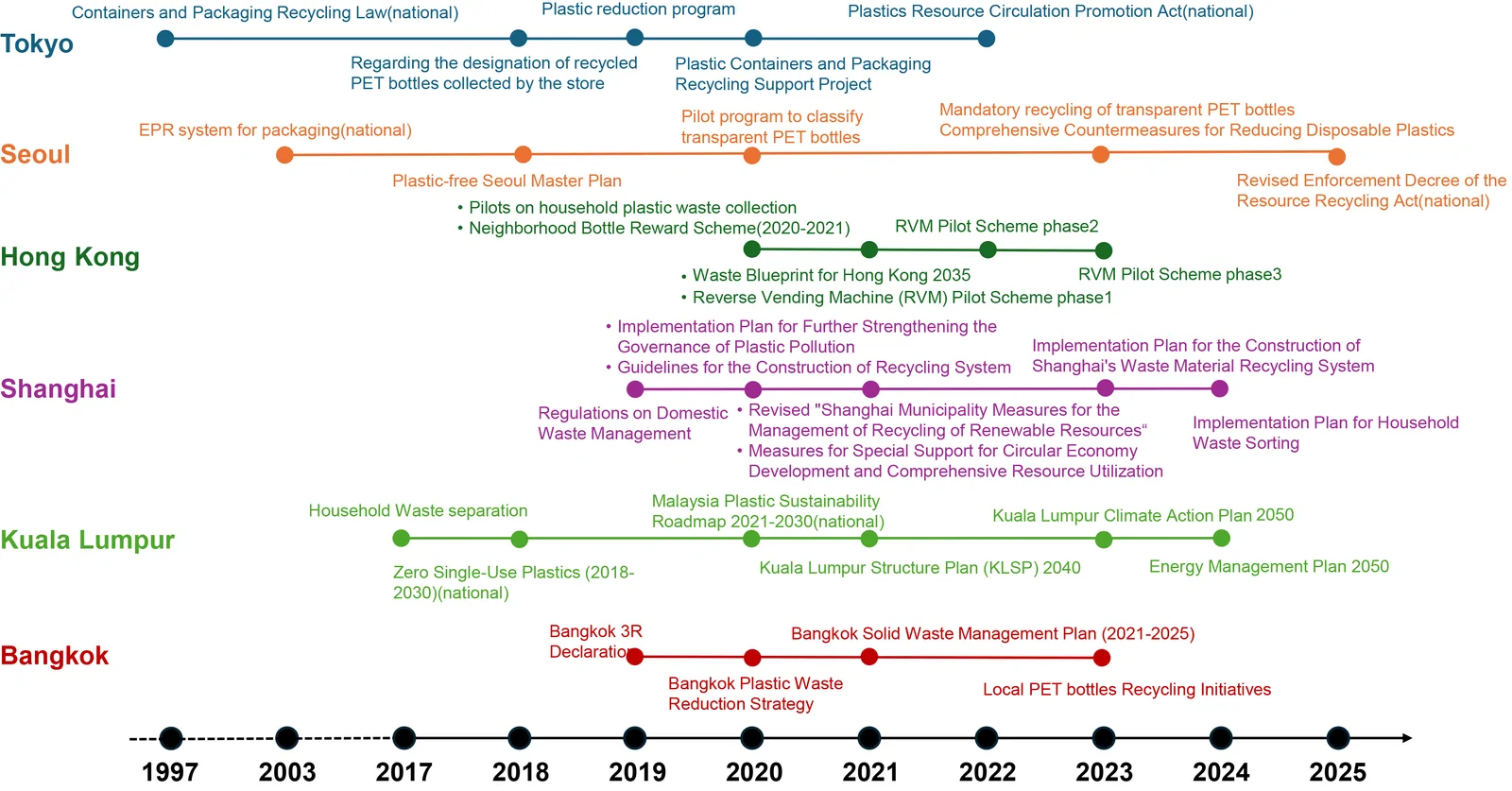
Policies and regulations related to PET bottle recycling in six cities The horizontal axis represents the year, while different colors indicate the policies and regulations implemented in each city.

The infrastructure dimension further distinguishes mature institutional systems from emerging or fragmented recovery systems. Tokyo and Seoul have relatively integrated municipal infrastructure that links source separation, collection, transfer, intermediate treatment, and downstream recycling. In Tokyo, municipalities collect discarded PET bottles separately and transport them to intermediate facilities, where bottles are washed, shredded, and then allocated to recycling enterprises through a bidding mechanism.^78^ In-store take-back schemes and designated recycling channels further strengthen the supply of recyclable PET to domestic processors.^60^ Seoul has expanded transparent PET bottle recovery through apartment-based collection systems, dedicated collection for detached housing areas, and subsidized unmanned collection machines in community centers, parks, public institutions, and other high-traffic locations.^79^^,^^80^ However, infrastructure availability has not yet translated into proportionate recovery performance, mainly because public participation and convenience remain limiting factors. Shanghai’s infrastructure is increasingly shaped by enterprise participation and platform-based services.^81^ Transfer stations and aggregation sites have been incorporated into municipal land-use planning, while smart collection machines, mobile collection stations, digital information platforms, and specialized sorting and processing facilities have improved system coordination. Bangkok and Kuala Lumpur still have limited infrastructure. Bangkok’s recovery infrastructure is still strongly dependent on informal collectors and non-standardized collection channels, although RVMs and digital services have begun to improve front-end recovery.^82^^,^^83^ Kuala Lumpur’s formal door-to-door collection system remains limited in coverage and capacity. Consequently, self-delivery to collection points and sales to scrap dealers continue to form the backbone of PET bottle recovery.^84^ Weak source-separation facilities and low household participation further reduce the quality and stability of the recovered PET feedstock.^18^ Detailed information on recycling facilities is provided in Table S1.

Taken together, Tokyo and Seoul exhibit the strongest institutional alignment among the six cases. Mandatory source separation, EPR, municipal coordination, and quality-oriented collection and recycling infrastructure mutually reinforce one another, creating relatively standardized front-end collection systems and stable supplies of higher-quality PET feedstock. These two cities can therefore be classified as having a high-level municipal intervention, as shown in Table S8. Hong Kong and Shanghai occupy an intermediate position. Hong Kong has established a visible government-supported recovery network through community recycling facilities, incentive schemes, and planned producer responsibility arrangements, but its system remains constrained by voluntary participation and insufficient local feedstock. Shanghai, by contrast, represents a transitional model in which a market-led recovery system is being progressively formalized through policy support, enterprise participation, and digital infrastructure. In both cities, municipal intervention supports PET bottle recovery either directly through public collection networks or indirectly through institutional coordination and market formalization. They can therefore be classified as having a medium-level municipal intervention. Bangkok and Kuala Lumpur remain more dependent on informal and market-mediated recovery networks. Although the residual material value of PET bottles continues to sustain collection activities, weak enforcement, limited infrastructure, unstable source separation, and uneven front-end quality control constrain the overall system performance. These two cities are therefore classified as having a low-level municipal intervention.

### Corporate behavior and recycling market dynamics

Corporate behavior and recycled PET market conditions further shape the efficiency, quality, and value of PET bottle recycling across the six cities. These factors operate through upstream packaging design, enterprise-based collection networks, recycling technologies, downstream demand, and market-based incentives, determining whether post-consumer PET bottles can be rapidly captured, upgraded, and reintegrated into material value chains. Table S2 compares corporate practices in PET bottle recycling across the six cities.

Packaging producers are upstream actors in the PET bottle value chain, and their design choices strongly affect downstream sorting, material purity, and recycling efficiency. Tokyo and Seoul show the clearest progress in this respect. In Tokyo, voluntary design guidelines have standardized PET bottle design and improved recyclability,^59^ while major beverage companies, including Suntory,^85^ Coca-Cola,^86^ and Kirin,^87^ have advanced horizontal recycling, recycled PET use, and dedicated collection infrastructure.^88^^,^^89^ Seoul has followed a similar trajectory through lightweighting,^90^ label-free bottles,^91^ laser marking, and the use of 100% recycled PET in municipal bottled water.^92^^,^^93^ These design interventions reduce contamination at source and improve sorting and reprocessing efficiency. In Shanghai, several leading local beverage companies have also taken initial but important steps toward source-level design improvement, including lightweight bottles, label-free packaging,^94^ fully recyclable packaging targets,^95^ and consumer-facing recycling campaigns.^96^^,^^97^^,^^98^ Although their current market share remains limited, these early actions provide visible examples for other beverage brands and indicate a gradual shift toward design-for-recycling practices. By contrast, upstream packaging design remains less active in Hong Kong, Bangkok, and Kuala Lumpur. In these cities, design-for-recycling has not yet been strongly reinforced by mandatory regulation, industry-wide producer responsibility, or stable demand for high-quality closed-loop recycled PET. In some cases, reliance on imported plastic waste also further reduces the incentive for packaging producers to invest in scalable recyclable design.^99^

Efficient PET recovery depends on enterprise networks that can rapidly capture, aggregate, and transfer bottles before material quality deteriorates. Shanghai provides a clear example of a market-driven enterprise recovery network. Since China’s plastic waste import ban, the sharp decline in imported waste PET feedstock has increased the demand for domestic post-consumer PET bottles, turning them into high-value secondary resources.^100^ This supply-demand imbalance has strengthened market incentives and accelerated the expansion of enterprise-led collection channels, including registered recycling enterprises,^69^ smart recycling bins,^70^ door-to-door services, fixed-source collection, and mobile collection stations.^54^ Together, these channels shorten the post-consumption collection cycle and help sustain Shanghai’s high PET bottle recovery rate. Tokyo and Seoul represent a different pathway, where enterprise participation is more strongly embedded in producer-responsibility-driven recycling networks.^40^^,^^101^ In these systems, beverage producers, packaging manufacturers, recycling organizations, and downstream recyclers are connected through formal EPR-based arrangements, which supports more institutionalized commercial recovery channels. In Hong Kong, Bangkok, and Kuala Lumpur, corporate and market-based collection channels have also expanded through RVMs,^46^ incentive schemes,^64^ company-supported collection programs,^102^ and public engagement platforms.^103^ However, these systems remain less integrated. Hong Kong has developed incentive-based recovery channels and is in the process of establishing an EPR framework for plastic beverage containers,^104^ but it is still constrained by insufficient upstream collection ability.^105^ Bangkok has active recycling firms and pilot collection schemes,^106^^,^^107^ although the wider deployment of RVMs remains limited by economic feasibility.^74^ Kuala Lumpur’s value chain is more fragmented, with local feedstock quality weakened by contamination, weak source separation, and continued reliance on imported inputs.^18^

Recycling technology and downstream market demand determine whether recovered PET bottles are converted into low-value secondary products or reintegrated into higher-value material cycles. Tokyo and Seoul are gradually moving beyond conventional mechanical recycling toward higher-value pathways, including bottle-to-bottle recycling,^108^ chemical recycling,^109^^,^^110^ biological recycling,^111^ AI-enabled sorting, and data-driven optimization of collection systems.^112^ Shanghai also has strong technological and industrial potential, supported by its petrochemical base, chemical recycling development, and emerging enzymatic PET recycling.^113^^,^^114^^,^^115^ However, Shanghai’s recycled PET market remains strongly oriented toward open-loop applications, especially recycled fibers for the textile sector.^116^ Strong fiber-sector demand provides a stable outlet for recovered PET bottles, but the high cost and limited scalability of chemical recycling make downcycling a more attractive option for many recyclers. Bangkok follows a market-oriented pathway dominated by mechanical recycling.^82^ Large recycling enterprises, particularly Indorama Ventures,^117^ process recovered PET bottles into flakes for fiber, sheet, and bottle-grade applications, while price structures indicate strong market value for recovered PET.^118^^,^^119^ To improve source-separated PET quality, Bangkok has piloted RVMs and other incentive-based schemes, but wider deployment remains constrained by weak economic feasibility.^120^ Hong Kong has taken a different route, with a food-grade PET recycling facility established through multi-stakeholder investment and support.^121^ The city has developed PET chemical recycling capacity, but facility operation is mainly constrained by insufficient local feedstock caused by weak upstream collection systems and low public participation.^122^ Kuala Lumpur also depends largely on mechanical PET recycling,^123^ but poor upstream collection and contaminated local feedstock often lead to downcycling, while recyclers tend to prefer higher-quality imported plastic waste over unstable domestic PET bottle supplies.

Overall, Tokyo, Seoul, and Shanghai show relatively high enterprise and market activity, but their strengths differ. Tokyo and Seoul benefit from stronger design-for-recycling practices, producer engagement, and higher-value recycling pathways. Shanghai’s advantage lies in its dense recycling enterprise network, extensive collection points and recycling facilities, and strong downstream demand for recycled PET. These enterprise-led collection channels enable discarded PET bottles to be rapidly captured at the source before they enter mixed waste streams, thereby supporting high recovery rates. Hong Kong and Bangkok occupy an intermediate position, with emerging corporate initiatives and market mechanisms but persistent constraints in upstream collection, local processing, facility utilization, and standardization. Kuala Lumpur remains comparatively weaker, reflecting limited enterprise participation, lower domestic feedstock quality, weaker advanced recycling capacity, and fragmented market integration. This weaker market activity is also partly associated with the inflow of imported plastic waste, which may reduce incentives to strengthen local PET bottle collection and constrain the development of a robust domestic recycling system.

## Context-sensitive lessons and collaboration opportunities

### Challenges and opportunities for six cities

#### Government action in bottle-to-bottle recycling

Government actions aimed at promoting bottle-to-bottle recycling play an important role in advancing high-value PET circularity across Asian cities, although the extent of policy intervention varies significantly. In Tokyo and Seoul, governments have introduced recycled-content mandates and related regulatory measures to stimulate closed-loop PET recycling. Tokyo requires beverage PET bottles to contain at least 15% recycled plastic by 2026 and has introduced packaging design standards to improve recyclability and material purity.^124^ Similarly, Seoul has adopted a gradual policy pathway requiring beverage producers using more than 5,000 tons of PET annually to incorporate at least 10% recycled plastic into their bottles from 2026.^125^

In contrast, other cities remain in earlier stages of policy development for bottle-to-bottle recycling. In Shanghai, although PET recovery rates are relatively high, the absence of unified regulatory standards governing recycled plastics in food-contact packaging constrains the development of closed-loop recycling and limits incentives for producers to adopt bottle-to-bottle production systems.^126^ However, the already high collection rate provides a strong material base for future policy development once regulatory standards are clarified.

The Hong Kong government has shown increasing support for plastic bottle recycling, and some enterprises have initiated bottle-to-bottle recycling activities. Nevertheless, the limited supply of post-consumer PET remains a key challenge, as front-end collection capacity is insufficient to support stable downstream recycling operations. In recent years, potential cross-boundary cooperation with southern Guangdong has been discussed,^127^ which may create opportunities for the regional integration of recycling infrastructure and more stable feedstock supply. In Bangkok and Kuala Lumpur, policy priorities currently focus on improving overall PET bottle recovery rates rather than promoting bottle-to-bottle recycling. Governments are therefore concentrating on strengthening waste management regulation, improving collection systems, and increasing investment in recycling infrastructure to build the foundation for higher-value recycling pathways.^18^^,^^128^

#### System structure and operational constraints

Institutional coordination and infrastructure capacity continue to constrain the efficiency of PET recycling systems across many Asian cities. In relatively advanced systems such as Tokyo and Seoul, challenges mainly arise from institutional coordination and the integration of recycling infrastructure. In Tokyo, the existing auction-based allocation mechanism may create mismatches between allocated recycling volumes and the actual processing capacity of recycling enterprises, as companies often submit bids under incomplete information regarding material quality and supply stability.^129^ Improving information transparency could therefore enhance the efficiency of material allocation across recycling enterprises. In Seoul, the lack of a fully integrated recycling infrastructure, together with variations in collection arrangements across residential types and administrative districts, complicates system coordination and limits the expansion of high-quality recycling pathways such as bottle-to-bottle systems.^125^ Strengthening coordination across districts and improving infrastructure integration could therefore support the development of more stable closed-loop recycling systems.

In Shanghai, the recycling industry is largely dominated by small- and medium-sized enterprises relying on conventional sorting and processing technologies, which constrain technological upgrading and the development of higher-value recycling pathways. At the same time, the relatively low entry barriers of the recycling sector have encouraged broad participation from small collectors, traders, and processing enterprises, forming a dense grassroots collection network. This extensive participation helps sustain high recovery rates of PET bottles and provides a flexible foundation for future system upgrading and value-chain integration. Hong Kong faces severe land scarcity and insufficient front-end collection capacity, both of which restrict the expansion of large-scale recycling facilities.^127^ These constraints may also create opportunities for regional cooperation. Bangkok experiences systemic inefficiencies due to the absence of an integrated solid-waste management framework, where PET bottles are frequently mixed with other waste streams during collection and transfer.^130^ Kuala Lumpur faces similar challenges, as fiscal pressure on the waste management system constrains the development of recycling infrastructure.^18^ These conditions highlight opportunities for improving operational coordination, optimizing collection and sorting processes, and enhancing the overall structural efficiency of PET recycling systems in these cities.

#### Integration and formalization of recycling networks

In market-driven informal recycling networks, the composition and activities of collection actors play a central role in shaping the performance of urban PET recycling systems. In cities such as Bangkok and Kuala Lumpur, recycling networks rely heavily on informal communities, where waste pickers, street collectors, and neighborhood junk shops form the backbone of PET bottle recovery. These decentralized networks operate largely based on market transactions and personal trust relationships.^18^^,^^130^ Although they provide extensive grassroots collection capacity and play an essential role in diverting plastic waste from landfills, informal recyclers often lack institutional recognition, stable income protection, and access to formal waste management systems.

Shanghai may provide a useful transitional reference for these cities, but its experience should not be treated as a directly transferable model. The partial formalization of Shanghai’s market-driven recycling system has relied on several enabling conditions, including dense collection and recycling infrastructure, digital or platform-based recycling services, stronger municipal coordination, waste-sorting enforcement, and stable downstream demand for recycled materials. These conditions allow small-scale recycling enterprises to bridge informal collection networks and formal municipal governance, reducing excessive reliance on individual waste pickers while retaining flexible collection capacity. For Bangkok and Kuala Lumpur, the key lesson is therefore not to replicate Shanghai’s pathway, but to assess whether similar institutional, infrastructural, and enforcement conditions exist or can be gradually developed. Where such conditions remain weak, a more incremental strategy is needed, such as mapping and registering informal collectors, supporting small recycling enterprises, improving source separation, and linking grassroots collectors with downstream recyclers. This context-sensitive approach could preserve the strengths of informal networks while improving material quality, operational efficiency, income security, and system sustainability. Figure 3 provides a visual summary of the key characteristics, strengths, and challenges of recycling systems across the six cities.

**Figure 3:**
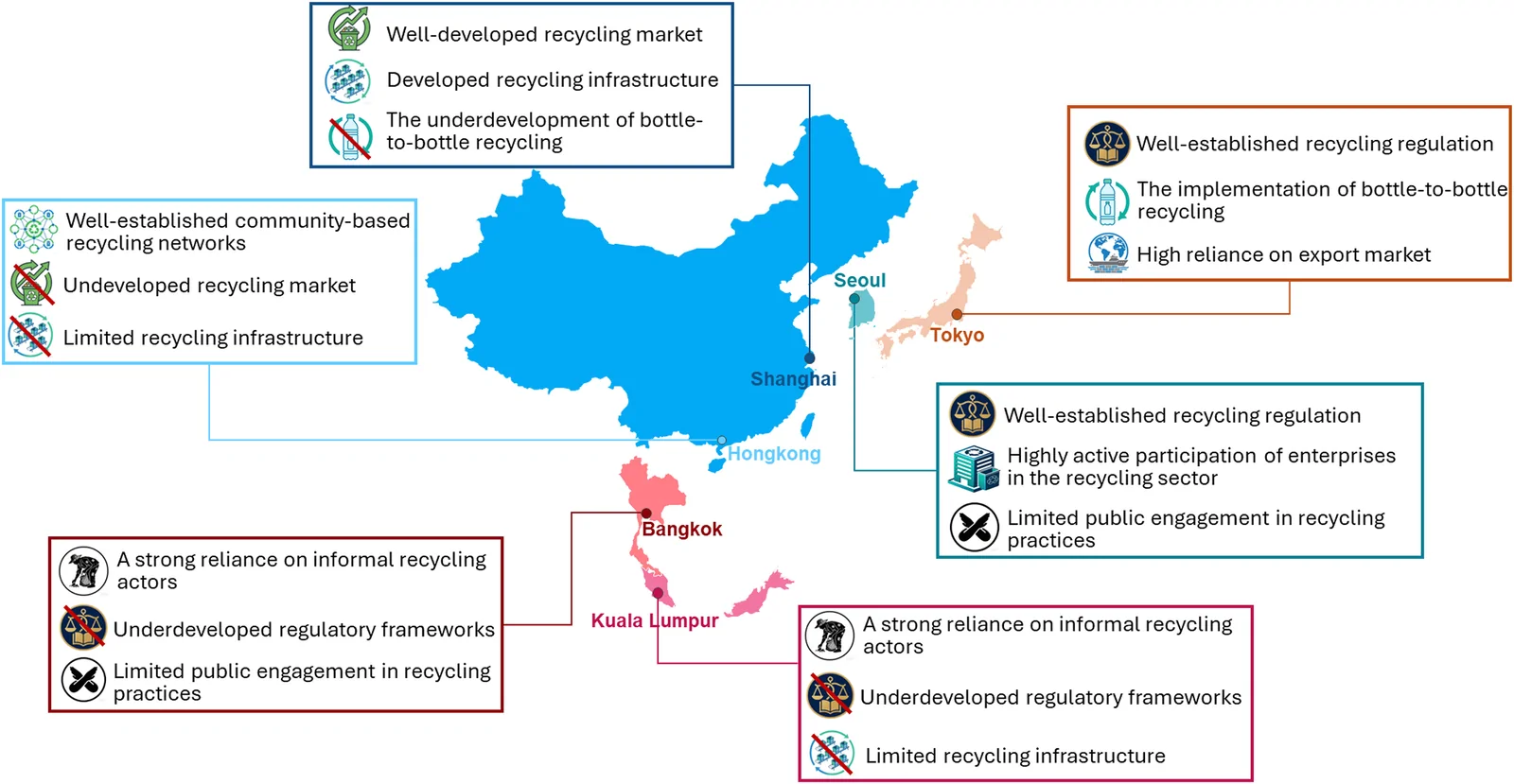
Key characteristics, strengths, and challenges of recycling systems across the six cities The figure summarizes the major strengths and challenges of PET bottle recycling systems in Tokyo, Seoul, Shanghai, Hong Kong, Bangkok, and Kuala Lumpur, including regulatory frameworks, recycling markets, infrastructure, enterprise participation, and informal recycling activities.

### Insights on successful recycling systems for other cities in Asia

#### Source separation as a prerequisite for high-quality waste PET bottle recycling

In cities where market-driven incentives are weak, waste recycling relies primarily on government regulation, making source separation a necessary condition for achieving high-quality PET bottle recovery. The finer the degree of separation at the source, the higher the purity of collected PET bottles, which in turn enhances the efficiency and value of downstream recycling processes. Experiences from Tokyo and Seoul demonstrate that strict and well-defined source separation plays a foundational role in the effective operation of their PET bottle recycling systems. By contrast, in cities where PET bottle recycling is largely driven by market mechanisms, insufficient front-end separation often results in contamination during collection and transportation. Consequently, a substantial share of recovered PET bottles is downcycled into lower-value products, reducing both recycling efficiency and material quality. As these limitations have become increasingly evident, many such cities have gradually shifted their policy focus toward front-end separation, recognizing its importance in alleviating the burden on downstream sorting and reprocessing. Therefore, source separation should be regarded as an essential component of PET recycling systems. In the initial stages of implementation, mandatory separation requirements, supported by strict enforcement, are necessary to ensure compliance, as reliance on voluntary public participation alone is unlikely to yield consistent outcomes. At the same time, systematic public education on waste classification is required. Once source separation becomes habitual, regulatory intensity may be gradually reduced. In addition, accountability mechanisms—such as those implemented in Shanghai—can be introduced to further enhance policy effectiveness and enforcement capacity.

#### The critical role of communities in promoting environmental awareness and household recycling practices

In many cities, the role of communities has often been underestimated during the implementation of recycling programs, with outreach efforts primarily targeting young people and students. However, empirical evidence suggests that communities play a significant and often unexpected role in disseminating environmental knowledge and mobilizing residents to participate in PET bottle recycling.^131^ The experience of Kuala Lumpur illustrates that community-based communication and information sharing among neighbors generate positive effects within PET bottle recycling networks.

Accordingly, community-level engagement should be prioritized when promoting environmental awareness and encouraging recycling behaviors. The effectiveness of such engagement is shaped by local social structures. In many East Asian cities, dense residential estates, property management systems, neighborhood organizations, and community recycling facilities provide organized channels for circulating recycling information and practices.^132^ In Southeast Asian cities, community mobilization often relies more strongly on neighborhood ties, informal collectors, waste banks, and community-based recycling networks.^133^ Within these contexts, retired residents and homemakers can play a particularly important role because they tend to have a stronger daily presence in local communities, wider interpersonal connections, and more frequent involvement in household routines.^134^^,^^135^ In particular, homemakers are often closer to the point where household recyclables are generated and separated, as they are more directly involved in daily practices such as purchasing, cleaning, storage, waste sorting, and preparation of recyclable materials for collection.^136^ Leveraging these community- and household-level actors can substantially enhance the reach and practical impact of recycling policies and related initiatives.

#### EPR as an effective instrument under mature legislative and regulatory frameworks

In practice, governments in Tokyo and Seoul have benefited from national EPR schemes, which have significantly eased the administrative and financial pressures associated with PET bottle recycling management. Hong Kong is also in the process of considering similar arrangements, largely in response to increasing fiscal constraints. However, the effective implementation of EPR places high demands on legislative clarity and regulatory capacity. Responsibilities across different actors must be clearly defined, accompanied by detailed operational guidelines, and supported by robust enforcement mechanisms. For many developing cities in Asia, these institutional prerequisites are not yet fully in place, making the immediate adoption of EPR less feasible. In such contexts, priority should instead be given to improving collection rates and ensuring the cleanliness and quality of collected materials before advancing toward more complex responsibility-sharing frameworks.

#### The government’s leading and coordinating role

Based on the experiences of the six cities examined, governments play an indispensable role in promoting PET bottle recycling systems. This role is reflected in policy guidance, support for and collaboration with recycling enterprises, and efforts to raise public awareness of recycling practices. Every stage of the recycling process relies on active government involvement. Therefore, improving the circular utilization of PET bottles requires governments to take the lead, guiding both enterprises and consumers to contribute to the system. Equally important is governmental credibility, which constitutes a fundamental condition for the long-term stability of recycling systems. The experiences of these cities indicate that once public trust in government is undermined—for example, when residents properly separate waste at the source but materials are subsequently mixed during collection or transportation—public motivation to participate in recycling is significantly weakened. Moreover, rebuilding trust after such failures is both difficult and costly, substantially increasing the challenges of sustained public engagement.

## Discussion

Under different urban governance contexts, post-consumer PET bottles are incorporated into distinct recovery systems. Tokyo, Seoul, and Hong Kong mainly rely on government-led municipal waste management systems, whereas Shanghai, Bangkok, and Kuala Lumpur are more strongly shaped by market-oriented recycling and secondary resource recovery networks. Although some cities show comparable PET bottle recycling rates, their institutional arrangements and operational mechanisms differ substantially. Tokyo and Seoul demonstrate the importance of refined source separation, producer collaboration, recyclability-oriented packaging design, and long-term environmental education. Shanghai highlights the role of a mature secondary resource market, dense recycling enterprise networks, and strong downstream demand in sustaining high PET bottle recovery. Bangkok and Kuala Lumpur, by contrast, show the continued importance of informal collectors, community networks, and pilot initiatives in expanding PET bottle recovery under more constrained governance conditions.

These findings show that there is no single pathway to PET bottle circularity in Asia. Policy learning should therefore be context sensitive rather than based on direct replication. More mature systems can provide useful references for improving source separation, material purity, producer responsibility, and public participation, but their applicability depends on local institutional capacity, infrastructure, enforcement, market maturity, and social conditions. For cities with more fragmented or market-driven systems, the immediate priority may be to strengthen collection coverage, recognize and integrate informal actors, improve front-end separation, and build stable links between collectors and downstream recyclers. Overall, Asian cities should build on their own governance and market strengths while selectively adapting relevant experiences from others to improve PET bottle circularity and resource recovery.

### Limitations of the study

This study has several limitations. City-level PET bottle recycling data are unavailable for some cities, requiring national average recycling rates to be used as proxies, which may not fully reflect local conditions. In addition, the assessment framework mainly considers government interventions and enterprise participation, with limited coverage of consumer behavior and informal recycling activities. Differences in statistical definitions and reporting practices may also affect cross-city comparability. Future research should develop more granular and standardized city-level datasets and integrate policy, enterprise, technological, infrastructural, market, and social dimensions to provide a more comprehensive and robust assessment of PET bottle recycling systems.

## Resource availability

### Lead contact

Requests for further information and resources should be directed to and will be fulfilled by the lead contact, Xuemei Zhang (xuemei.zhang@nottingham.edu.cn).

### Materials availability

This study did not generate new unique reagents.

### Data and code availability

• All data used in this study were obtained from publicly available municipal policy documents, government reports, industry statistics, corporate sustainability reports, academic literature, and publicly accessible information on PET bottle recycling systems. The study did not generate proprietary datasets or require specialized experimental resources.
• This paper does not report original code.
• Any additional information required to reanalyze the data reported in this paper is available from the lead contact upon request.

## Acknowledgments

This work was founded by the 10.13039/501100001809National Natural Science Foundation of China (nos. 72525006, W2432029, and 41850410497).

## Author contributions

X.Z., proposed the outline, organized the manuscript, and contributed to formal analysis and writing – original draft preparation; F.K.S.C., conceptualization, writing – reviewing and editing, and supervision; Y.T., data curation, investigation, and formal analysis; Yuyao Xu, validation and writing – review and editing; C.W., validation and writing – review and editing; M.J., validation and writing – review and editing; T.N., validation and writing – review and editing; Yaoyang Xu, validation and writing – review and editing; Y.-G.Z., validation and writing – review and editing; W.-Q.C., validation, writing – reviewing and editing, and supervision.

## Declaration of interests

The authors declare no competing interests.

## Declaration of generative AI and AI-assisted technologies in the manuscript preparation process

During the preparation of this manuscript, the authors used ChatGPT (GPT-5.4) to assist with language refinement. All ideas, methodologies, experiments, and conclusions were developed and verified by the authors.

## STAR★Methods

### Key resources table

**Table:** 

REAGENT or RESOURCE	SOURCE	IDENTIFIER
**Deposited data**		

Municipal policy and regulatory documents	Municipal governments	https://www.petbottle-rec.gr.jp/ https://pr-bangkok.com/ https://www.shhk.gov.cn/ https://www.wastereduction.gov.hk/en-hk
Government statistics and reports	National and municipal statistical agencies	https://www.kankyo.metro.tokyo.lg.jp/ https://www.nso.go.th/ https://www.jcpra.or.jp/ https://www.shhuangpu.gov.cn/ https://www.info.gov.hk/ https://news.seoul.go.kr/
Industry statistics and reports	Relevant industry associations and organizations	https://bottledwater.org/ https://www.danone.com.cn/ https://www.suntory.com/
Corporate sustainability reports	Relevant companies	https://www.unescap.org/ https://www.unep.org/
Peer-reviewed literature	Academic publications	https://scholar.google.com/

**Software and algorithms**		

Microsoft Excel	Microsoft	https://www.microsoft.com/
Microsoft Visio	Microsoft	https://www.microsoft.com/
Adobe Illustrator	Adobe	https://www.adobe.com/

### Method details

#### Study design and data collection

This study adopted a structured qualitative comparative approach to examine PET bottle recycling systems across six Asian cities. Information was collected on municipal policies, recycling infrastructure, collection practices, producer responsibility, recycling technologies, market activities, and corporate practices. Because city-level PET bottle recycling data are not consistently reported using harmonized definitions, the analysis did not rely solely on quantitative recycling indicators. Instead, the six cities were compared based on their institutional arrangements, municipal interventions, enterprise participation, and recycling market characteristics.

#### Data quality assessment

Consistent with previous studies,^137^^,^^138^ a data quality assessment framework was established (Table S3) to evaluate data uncertainty. Five indicators were used to characterize major sources of uncertainty, including source reliability, temporal correlation, geographical correlation, and methodological transparency. Each indicator was assigned a score of 0, 0.5, or 1 according to explicit scoring rules, and an overall data quality score was calculated using Equation 1, with higher scores indicating better data quality. When multiple studies reported different values for the same metric (e.g., recycling rate) for the same region, the value associated with the highest overall data quality score was selected. All data extraction and evaluation procedures were conducted consistently based on the predefined criteria to ensure transparency and reproducibility. Priority was given to official municipal documents, government reports, peer-reviewed studies, industry statistics, and corporate sustainability reports. Recent and city-specific data on PET bottles or plastic beverage containers were preferred over broader national-level plastic waste data. Where multiple sources reported similar indicators, the information was cross-checked to identify the most consistent and reliable estimate.

Source reliability (SR): This dimension assesses the credibility and authority of the data source, with higher scores assigned to official statistics, government reports, producer responsibility organizations, industry associations, and peer-reviewed studies.

Temporal correlation (TC): This dimension pertains to the consistency between the available data and the ideal temporal reference point, ensuring that the data aligns with the specified time frame.

Geographical correlation (GC): This dimension refers to the consistency between the available data and the ideal geographical reference point, ensuring that the data corresponds accurately to its geographical origin.

Material representativeness (MR): This dimension evaluates whether the reported data specifically reflect PET bottles rather than broader categories such as PET packaging, plastic bottles, general plastics, or municipal solid waste.

Methodological transparency (MT): This dimension examines whether the definition, calculation method, system boundary, and statistical scope of the reported PET bottle recycling rate were clearly described.

The Total Score (TS) was calculated based on the above indicators, representing a comprehensive assessment of data quality. As expressed in Equation 1, TS ranges from 0 to 1, with a score value closer to 1 indicating higher data quality, while nearer to 0 indicating lower quality.

(Equation 1) TS=SR∗0.2+TC∗0.2+GC∗0.2+MR∗0.2+MT∗0.2

Based on the scoring results in Table S4, the data point with the highest total score was selected as the representative PET bottle recycling rate for each city. The selected values for Tokyo, Seoul, Shanghai, Hong Kong, Bangkok, and Kuala Lumpur were 92%, 79%, 95.4%, 14%, 52%, and 23%, respectively. Lower-scoring values were not directly adopted but were retained for uncertainty analysis and cross-validation.

Cross-validation was conducted by comparing the selected value with other available estimates for the same city or country. Values with similar scores and comparable numerical ranges were used to assess the consistency of the selected estimate. For example, Seoul showed highly consistent reported values ranging from 79% to 80%, while Shanghai showed close estimates of 95.4% and 96.6%. Bangkok’s selected value of 52% was also broadly comparable with the alternative estimate of 62%. In contrast, larger discrepancies for Hong Kong and Kuala Lumpur mainly reflected differences in data scope, material coverage, or calculation boundaries, and were therefore interpreted with greater caution. Overall, the selected values were supported by both the highest quality scores and cross-source consistency, indicating that they were adopted on a transparent and evidence-based basis.

#### Analytical framework

The analytical framework consisted of two interconnected modules: (1) recycling system and municipal intervention and (2) corporate practices and recycling market. Each module comprised four analytical dimensions. The framework was used to systematically assess the organization and operation of PET bottle recycling systems across the six cities (Table 2).

#### Assessment of municipal intervention and enterprise participation

To support a structured qualitative comparison, two complementary scoring schemes were used to evaluate PET bottle recycling systems. Effective municipal intervention was assessed across seven criteria: policy framework, implementation and enforcement, municipal responsibility, source-separation support, infrastructure support, and stakeholder coordination. Enterprise participation and recycled PET market activity were assessed across six criteria: packaging design support for recycling, formal recycling enterprise network, collection and aggregation capacity, recycling technology capacity, downstream demand for recycled PET, and market-based collection incentives and participation. For both evaluations, each criterion was scored as 1, 0.5, or 0, representing strong, partial, or weak evidence, respectively.

These scores were used to support structured comparison rather than to produce an absolute ranking of city performance. Higher municipal intervention scores indicate stronger and more effective public governance, while higher enterprise activity scores indicate stronger enterprise support for PET bottle recovery. However, these indicators should not be interpreted as direct measures of overall recycling system effectiveness, as government-led and market-driven systems may achieve recycling performance through different mechanisms. The scoring results for effective municipal intervention and enterprise participation are presented in Tables S7 and S8, respectively.

### Quantification and statistical analysis

The predefined assessment criteria were used to assign scores for municipal intervention and enterprise participation in each city. Scores were determined according to the evidence identified during the data collection and quality-assessment process. The resulting scores were used to support the comparative assessment of PET bottle recycling systems rather than to establish a universal ranking of city performance. All empirical analyses were performed using Microsoft Excel for Microsoft 365 MSO (Version 2608, Build 16.0.20326.20072) 64-bit. Figures were created using Microsoft Visio, PowerPoint, and Adobe Illustrator, and tables were prepared using Microsoft Excel.

### Additional resources

No additional specialized resources were required for this study.
