# Supplementary material for: PET bottle recycling system in Asia: Lessons learned from six cities
Source: iScience. 2026 Nov 2;29(11):118095. doi: 10.1016/j.isci.2026.118095 (PMC13756065; doi:10.1016/j.isci.2026.118095)
Supplement: Supplementary file 1 [file mmc1.pdf]

iScience, Volume *29*

Supplemental information

PET bottle recycling system in Asia: Lessons learned from six cities

Xuemei Zhang, Faith Ka Shun Chan, Yuanyuan Tang, Yuyao Xu, Chao Wang, Matthew Johnson, Taiseer Nafea, Yaoyang Xu, Yong-Guan Zhu, and Wei-Qiang Chen

Table S1. Detailed comparison of PET bottle recycling systems in six cities, related to Figure 1 and Table 3.

City	Main PET bottles collector	Material recycling facilities	How to dispose and collect
Tokyo	Municipal government	The government is responsible for roadside recycling on designated days.	• Collect separately<br>• Remove the caps and labels<br>• Rinse the inside lightly with water<br>• Crush it<br>• Place in the green net bag at the collection site
Seoul	Municipal government	The government is responsible for roadside recycling on designated days.	• Collect separately<br>• Remove the labels<br>• Rinse the inside lightly with water
Hong Kong	Collection companies + Informal sectors	• 800 recyclable collection spots<br>• 7 transfer stations<br>• 245 collecting and recycling companies	Directly
Shanghai	Collection companies + Informal sectors	• 15,000 recyclable collection points<br>• 198 transfer stations<br>• 15 distribution centres	Directly
Bangkok	Informal sectors	• 289 recycling centres<br>• 3 transfer stations	Directly
Kuala Lumpur	Informal sectors	• 86 recycling centres<br>• 1 transfer stations	Directly

Table S2. Comparison of corporate practices across cities, related to Figure 1 and Table 3.

City	PET bottles design	r-PET content	Collection & Recycling	Governance & Role
Tokyo	• Lightweight<br>• Not coloured<br>• Label contains no polyethylene<br>• Inks do not transfer to PET bottles<br>• The closure is made mainly of PE/PP with a specific gravity less than 1.0	15% (mandate)	• Bottle-to-bottle recycling project<br>• Reverse vending machine<br>• Mechanical recycling<br>• Chemical recycling	• **PET bottles manufacturers & Users:** Pay the recycling fee<br>• **Recycling companies**: Provide the recycling service through government bidding
Seoul	• Lightweight<br>• Not coloured<br>• No label<br>• Easy-to-recycle tethered bottle caps and eco-friendly laser marking	10% (mandate)	• Bottle-to-bottle recycling project<br>• Reverse vending machine<br>• Mechanical recycling<br>• Chemical recycling	• **PET bottles manufacturers & Users:** Pay the recycling fee<br>• **Collection and recycling companies:** Implement recycling with government subsidies
Shanghai	• Lightweight<br>• Laser printing replaces ink<br>• Shrink bottle labels made of recycled materials	Prohibit	• Internet + recycling<br>• Collection facilities<br>• Reverse vending machine<br>• Mechanical recycling	• **Collection companies:** Decentralised collection and centralised sorting and processing<br>• **Recycling companies:** Convert the waste PET bottles to the r-PET
Hongkong	• Lightweight<br>• No label<br>• Not coloured	Individual companies voluntarily use 100% r-PET (No mandate)	• Reverse vending machine<br>• Collection from informal collectors<br>• Mechanical recycling<br>• Food-grade plastic recycling facility	• **Collection companies:** Implement the collection at government-established points<br>• **Recycling companies:** Convert the waste PET bottles to the r-PET
Kuala Lumpur	• Lightweight	Individual companies voluntarily use 100% r-PET (No mandate)	• Collection from informal collectors<br>• Mechanical recycling	• **Recycling companies:** Buying waste PET bottles from informal collectors and pre-reprocess

Bangkok	• Not coloured<br>• Lightweight<br>• Stop using ink in caps	Individual companies voluntarily use 100% r-PET (No mandate)	• Collection from informal collectors<br>• Mechanical recycling	• **Recycling companies:** Buying waste PET bottles from informal collectors and pre-reprocess

Table S3. Evaluation criteria of data quality, related to Table 3.

Indicator	Good (Score: 1)	Moderate (Score: 0.5)	Poor (Score: 0)
Source reliability (SR)	Data are from official statistics, government reports, producer responsibility organizations, industry associations, or peer-reviewed studies.	Data are from credible secondary sources, company reports, news reports, or reports that cite primary sources but do not provide full original data.	Data are from unclear, unverifiable, non-authoritative, or unsourced materials.
Temporal correlation (TC)	Data year is the same as the target year, or the time difference is within one year.	Data are from a nearby period, with a time difference of two to three years, and the recycling system is assumed to be relatively stable.	Data are outdated, with a time difference of more than three years, or the year of the data is not specified.
Geographical correlation (GC)	Data directly correspond to the target city.	Data are available at a broader or neighbouring spatial scale, such as national-level data used to approximate city-level conditions.	Data are from a different region, or the geographical scope is unclear and cannot represent the target area.
Material representativeness (MR)	Data specifically refer to PET bottles.	Data refer to PET packaging, plastic bottles, or beverage containers, and PET bottles are a major component.	Data refer only to general plastics, municipal solid waste, or packaging waste, without clear relevance to PET bottles.
Methodological transparency (MT)	The definition, calculation method, system boundary, and statistical scope of the recycling rate are clearly described.	The recycling rate is reported, but the calculation method or system boundary is only partly described.	The recycling rate is reported without any explanation of definition, calculation method, or statistical boundary.

Table S4. The result of uncertain analysis, related to Table3.

City	Data	SR	TC	GC	MR	MT	TS	Outcome
Tokyo	92%[1]	1	1	1	1	1	1	Selected
Tokyo	85.1%[2]	1	1	0.5	1	1	0.9	-
Seoul	79%[3]	1	1	0.5	1	1	0.9	Selected
Seoul	79%[4]	1	0.5	0.5	1	1	0.8	-
Seoul	80%[5]	1	0	0.5	1	0	0.5	-
Shanghai	95.4%[6]	1	1	0.5	1	1	0.9	Selected
Shanghai	96.6%[7]	1	0.5	0.5	1	1	0.8	-
Shanghai	90%	1	1	0.5	0	0	0.5	-

Hong Kong	14%[8]	1	1	1	1	0	0.8	Selected
Hong Kong	12.3%[9]	0.5	1	1	1	0	0.7	-
Hong Kong	33%[10]	1	1	0.5	0	0	0.5	-
Bangkok	52%[11]	1	0.5	1	1	1	0.9	Selected
Bangkok	62%[12]	1	0	0.5	1	1	0.8	-
Kuala Lumpur	23%[11]	1	0.5	1	1	1	0.9	Selected
Kuala Lumpur	28.06%[13]	1	0.5	0.5	0	1	0.6	-
Kuala Lumpur	62%[14]	1	0	0.5	0.5	0.5	0.5	-

Table S5. Criteria for evaluating policy intervention intensity, related to Table3.

Evaluation criterion	Score = 1	Score = 0.5	Score = 0
**Policy framework (PF)**	Clear and specific policies exist for PET bottle recycling, plastic packaging, source separation, or producer responsibility.	General waste or plastic policies exist, but PET bottle-related requirements are indirect or incomplete.	No clear policy framework related to PET bottle recycling or plastic bottle management.
**Implementation and enforcement (IE)**	Policies are actively implemented, monitored, and supported by enforcement mechanisms.	Policies are implemented in some areas or through pilot programmes, but enforcement is uneven.	Policies are weakly implemented, symbolic, or lack visible enforcement.
**Municipal responsibility (MR)**	Municipal authorities clearly organise, coordinate, or supervise PET bottle collection and recycling.	Municipal authorities provide partial coordination or support, but market or informal actors remain dominant.	Municipal involvement is very limited, and PET bottle recovery is mainly left to market or informal actors.
**Source-separation support (SS)**	PET bottles are collected separately from other waste streams, with clear rules or collection arrangements specifically for PET bottles or plastic beverage bottles.	Household waste is generally separated at source, but PET bottles are not collected as a distinct category and are usually grouped with other recyclables or plastics.	Waste is not mandatorily separated at the collection stage, and PET bottles are often collected together with mixed municipal waste or unsorted recyclables.
**Infrastructure support (IS)**	Collection points, transfer stations, sorting facilities, or recycling channels are well supported by public policies	Some infrastructure exists, but coverage, accessibility, or coordination remains limited	Publicly supported recycling infrastructure is weak or absent
**Stakeholder**	Policies effectively	Some coordination	No clear

**coordination (SC)**	coordinate governments, producers, recyclers, communities, and consumers.	exists, but responsibilities remain fragmented.	coordination mechanism among key actors.

Table S6. Scoring criteria for enterprise participation and recycling market activity across the PET bottle value chain, related to Table3.

Evaluation criterion	Score = 1	Score = 0.5	Score = 0
**Packaging design support for recycling (PD)**	Recyclable PET bottle design is widely adopted or clearly supported by policy/industry standards, such as lightweight design, colourless bottles, easy-to-remove labels, reduced ink use, or recycled-content design.	Some leading companies have begun to improve PET bottle design for recycling, and the industry is actively exploring recyclable packaging design, but adoption remains uneven and has not yet become a common practice.	Only isolated or very limited company-level attempts can be observed, while there is no clear policy support, industry-wide action, or systematic shift toward recyclable PET bottle design.
**Formal recycling enterprise network (RN)**	A dense and active network of formal collection, sorting, recycling, or recycled-material enterprises exists.	Some formal recycling enterprises exist, but the network is limited, uneven, or still developing	Formal recycling enterprises are few, weak, or not clearly involved in PET bottle recovery.
**Collection and aggregation capacity (CC)**	PET bottle collection, transfer, sorting, and aggregation are stable, accessible, and well connected.	Collection and aggregation exist but are fragmented, unstable, or dependent on limited channels.	Collection and aggregation capacity is weak or highly informal.
**Recycling technology capacity (RT)**	High-quality recycling capacity exists, such as advanced mechanical recycling, food-grade rPET, bottle-to-bottle recycling, or chemical recycling.	Basic mechanical recycling or pre-processing exists, but high-quality recycling capacity remains limited.	Recycling technology is weak or mainly limited to low-value processing.
**Downstream demand for recycled PET (DP)**	Strong downstream demand exists for recycled PET, including rPET flakes, pellets, fibres, packaging materials, or bottle-to-bottle applications. This demand creates clear economic incentives for PET bottle collection and recycling.	Downstream demand exists but is still emerging, unstable, limited to certain companies, pilot projects, or lower-value applications. It has not yet formed a strong system-wide pull for PET bottle recovery.	Downstream demand for recycled PET is weak, unclear, or poorly connected to the local PET bottle recycling system.
**Market-based collection**	PET bottles are actively recovered	Some market-based or incentive-based	Market-based participation is weak.

**incentives and participation (MP)**	through market incentives, such as buy-back channels, junk shops, mobile collectors, informal collectors, community-level trading, or platform-based recycling. Residents or local actors have clear economic incentives to collect, store, sell, or deliver PET bottles.	collection channels exist, but participation is uneven, small-scale, or limited to certain communities, pilots, or voluntary schemes.	PET bottles are mainly discarded through general waste channels or recovered only after disposal, with limited economic incentive for residents or local actors to participate.

Table S7. Scoring interpretation, related to Table3.

**Total score**	**Evaluation result**	**Interpretation**
4.0–6.0	High effective municipal intervention	Policies are well implemented and have clear influence on PET bottle recycling performance
2.0–4.0	Moderate effective municipal intervention	Policies exist and have partial effects, but implementation or system outcomes remain uneven
0–2.0	Low effective municipal intervention	Policies are absent, weakly implemented, or have limited observable influence on the recycling system

Table S8. Effective municipal intervention scores across six cities, related to Table3.

**City**	**PF**	**IE**	**MR**	**SS**	**IS**	**SC**	**Total**	**Level**
Tokyo	1	1	1	1	1	1	6	High
Seoul	1	0.5	1	1	1	1	5.5	High
Shanghai	0.5	1	0.5	0.5	1	0	3.5	Moderate
Hong Kong	0.5	0.5	1	0.5	0.5	0.5	3.5	Moderate
Bangkok	0.5	0	0	0.5	0.5	0	1.5	Low
Kuala Lumpur	0.5	0	0	0.5	0.5	0	1.5	Low

Table S9. Scores of stakeholder enterprise participation and recycling market activity, related to Table3.

**City**	**PD**	**RN**	**CC**	**RT**	**DP**	**MP**	**Total**	**Level**
Tokyo	1	1	1	1	1	0.5	5.5	High
Seoul	1	1	1	0.5	1	0.5	5	High
Shanghai	0.5	1	1	0.5	1	1	5	High
Hong Kong	0.5	0.5	0.5	0.5	0.5	0.5	3	Moderate
Bangkok	0.5	0.5	0.5	0	1	1	3.5	Moderate

Kuala Lumpur	0	0.5	0.5	0	0.5	0.5	2	Low

Reference

1. Council, P.B.R.P. (2025). Recycling Statistics. https://www.petbottle-rec.gr.jp/qanda/sec8.html.
2. Suishinkyo, P. (2024). Recycling Rate of PET bottles. https://www.petbottle-rec.gr.jp/english/actual2.html.
3. Lee, C., Jang, Y.-C., Choi, K., Kim, B., Song, H., and Kwon, Y. (2024). Recycling, Material Flow, and Recycled Content Demands of Polyethylene Terephthalate (PET) Bottles towards a Circular Economy in Korea. Environments *11*, 25. 10.3390/environments11020025.
4. Jang, Y.-C. (2021). Material and financial flows of plastic packaging recycling by EPR in Korea and the Philippines: potential gaps and suggestions. Chungnam National University.
5. SMG (2023). Seoul City to Implement Mandatory Separation of Transparent PET Bottles in Apartment Buildings Starting the 25th. https://news.seoul.go.kr/env/archives/510080.
6. Yingying, B., Jingyang, L., Li, D., and Xiaoming, S. (2022). Study on the production and recovery level of waste PET drinking bottles in China. Journal of Environmental Engineering Technology *12*, 185-190.
7. Jiameng, R., Feilong, Z., Li, D., and Jingyang, L. (2026). Generation and recycling of PET beverage packaging in China: current status and projections. Journal of Environmental Engineering Technology *16*, 9. 10.12153/j.issn.1674-991X.20250274shu.
8. ACE (2025). Common Legislative Framework for Producer Responsibility Scheme and the Producer Responsibility Scheme on Plastic Beverage Containers and Beverage Cartons. Advisory Council on the Environment.
9. Ng, E. (2024). How Hong Kong can rescue the struggling recycling industry and win its war on plastic waste. South China Morning Post.
10. EPD (2024). MONITORING OF SOLID WASTE IN HONG KONG Waste Statistics for 2023. https://www.wastereduction.gov.hk/sites/default/files/resources_centre/waste_statistics/msw2023_eng.pdf.
11. Cola, C. (2019). Full Circle: Accelerating the Circular Economy for Post-Consumer PET Bottles in Southeast Asia. Coca Cola.
12. Chairat, S., and Gheewala, S.H. (2023). Life cycle assessment and circularity of polyethylene terephthalate bottles via closed and open loop recycling. Environmental Research *236*, 116788. https://doi.org/10.1016/j.envres.2023.116788.
13. Pariatamby, A., and Barasarathi, J. (2021). EPR POLICY REVIEW REPORT. WWF.
14. Trisyanti, D., Wengi, K.R.L., Rachmawati, R.L., and Akib, R. (2022). ADVANCING THE PO ANCING THE POTENTIAL OF PE AL OF PET AND PP-BASED BEVERA T AND

PP-BASED BEVERAGE PACKAGING TO SUPPOR O SUPPORT CIRCUL T CIRCULAR ECONOM AR ECONOMY Journal of Environmental Science and Sustainable Development *5*, 25.
